# Association of Maternal Versus Fetal Ehlers-Danlos Syndrome Status with Poor Pregnancy Outcomes

**DOI:** 10.1007/s43032-022-00992-1

**Published:** 2022-06-08

**Authors:** L. A. Underhill, C. Barbarita, S. Collis, R. Tucker, B. E. Lechner

**Affiliations:** 1 Department of Pediatrics, Women and Infants Hospital of Rhode Island, Kilguss Research Institute, The Warren Alpert Medical School of Brown University, 101 Dudley Street, Providence, RI 02905 USA; 2 Brown University, Providence, RI USA

**Keywords:** Ehlers-Danlos syndrome, Pregnancy, Adverse outcomes, PPROM

## Abstract

The majority of research papers published on obstetrical outcomes in Ehlers-Danlos syndrome (EDS) have focused on the contribution of maternal EDS to the risk of poor pregnancy outcomes. The purpose of our study was to further clarify the fetal versus maternal contribution of EDS to poor pregnancy outcomes. A web-based, anonymous questionnaire was developed to collect pregnancy histories of families with a member with EDS. The survey was disseminated via social media through the Ehlers Danlos National Foundation. Population descriptors (age, gender, EDS diagnosis, age of diagnosis) and pregnancy descriptors (number of pregnancies, live births, and birth complications) were collected. To identify fetal and maternal contribution of EDS to poor pregnancy outcomes, three groups were compared based on maternal or infant diagnosis (EDS versus non-EDS). The rate of birth complications, treatment for preterm birth, and occurrence of preterm birth, as well as gestational age at preterm birth, were different depending on maternal/ infant EDS status, and these differences were significant when comparing infant EDS status but not when comparing maternal EDS status. The occurrence of PPROM is increased in the non-EDS mother/EDS infant group compared to both EDS mother/non-EDS infant and EDS mother/EDS infant groups (38.9%, 12.5%, 14.8%, *p* = 0.025). This study identifies that poor outcomes in EDS pregnancies differ depending on the maternal and the fetal EDS status. These insights into maternal and fetal association with certain poor pregnancy outcomes in pregnancies complicated by EDS can further guide physicians in educating, managing, and treating these women during pregnancy.

**Supplementary Information:**

The online version contains supplementary material available at 10.1007/s43032-022-00992-1.

## Background

The Ehlers-Danlos syndromes (EDS) are a clinically and genetically heterogeneous group of 13 heritable connective tissue disorders characterized by skin hyperextensibility, joint hypermobility, and tissue fragility [1]. The combined prevalence of all types of EDS is currently estimated to be 1 in 3500 to 1 in 5000 individuals worldwide [2].

The majority of research papers published on obstetrical outcomes in EDS have focused on the contribution of maternal EDS to the risk of poor pregnancy outcomes. These population-based studies, which include the most common maternal EDS types, identify a relationship between EDS and poor pregnancy outcomes [3–5]. When studies focused on single EDS types, the relationship between the EDS status of the patient and the risk of poor outcomes varied. For example, studies of patients with vascular EDS show an increase in the risk of poor pregnancy outcomes, while studies focused on patients with hypermobility EDS have not shown an increase in risk of pregnancy-related complications [6–11].

The first paper to associate poor outcomes in EDS pregnancies with the EDS status of the infant was published in 1966, prior to the first classification of EDS into types [12, 13]. In this case study of 18 EDS patients, 78% were reported to be born prematurely, while none of the healthy siblings of these premature babies was born premature. We have previously created a mouse model of EDS by genetically knocking out the proteoglycans decorin and biglycan, resulting in spontaneous preterm birth during pregnancy [14]. We have also identified that the fetal membrane morphology of pups with these genetic deletions show inconsistent thicknesses in their fetal membrane structure [15]. Since fetal membrane strength and function are directly dependent upon structural integrity, any morphological abnormalities may lead to weaknesses in the fetal membrane integrity, which in turn may lead to poor pregnancy outcomes such as preterm birth. Given that the fetal membranes are of fetal and not maternal origin, led us to hypothesize that the EDS status of the infant may be equally important in anticipating poor pregnancy outcomes in women with EDS.

We developed a survey to collect pregnancy outcome information for pregnancies involving mothers and/or infants with EDS to further clarify fetal and maternal status, and the association to poor pregnancy outcomes and birth complications.

## Methods

A web-based, interactive, anonymous questionnaire was developed on Survey Monkey to collect pregnancy histories of families with a member with EDS. An open, non-randomized format with branching components was employed, along with a comment section for respondents to clarify information given. The person responding to the survey is referred to as “respondent.” The respondent then answers questions regarding herself and her offspring. Generational data was combined into “mother/maternal” and “infant/baby/fetal.” The survey was disseminated via social media through the Ehlers-Danlos National Foundation. Responses were collected from March through May of 2016.

Our local ethics committee, the Women & Infants Hospital Institutional Review Board, ruled that no formal ethics approval was required for our survey, and as such, consent was not necessary. For exempt studies that were approved prior to 2019, as this study was, the applicable regulation falls under 45 CFR 46.101. The study was determined to qualify for exempt category b2.

Quantitative data collected included both population descriptors and pregnancy descriptors. Population descriptors such as age, gender, EDS diagnosis, and age of diagnosis were self-reported. Our exclusion criteria included surveys from male respondents, and repeat IP addresses. Pregnancy descriptors such as number of pregnancies, number of live births, and gestational age at birth were self-reported, while descriptors of ante-, intra-, and post-partum preterm birth complications were chosen from a list of subcategories. For this study, the poor pregnancy outcomes we focused on were birth complications, cesarean delivery, treatment for preterm birth (PTB), and occurrence of PTB, defined as a gestational age of less than 37w0d.

Data analysis was conducted on the continuous variable of gestational age using Kruskal–Wallis (three groups) and Wilcoxon (two groups) tests, due to the variable’s non-normal distribution. Categorical variables such as pregnancy/birth complications were analyzed with the chi-square test. The entire sample was analyzed first as three groups (EDS mother/EDS infant, EDS mother/non-EDS infant, non-EDS mother/ EDS infant), followed by subgroup analyses contrasting infant status into two groups (EDS or non-EDS) and maternal status into two groups (EDS or non-EDS) to determine the association between maternal and fetal status and poor pregnancy outcomes and complications associated with preterm delivery. Risk ratios and 95% confidence intervals (CI) were calculated for the 2 × 2 tables.

## Results

A total of 1704 responses were collected, representing 22 countries. A copy of the survey is presented in Supplemental File 1.

**Fig. 1:**
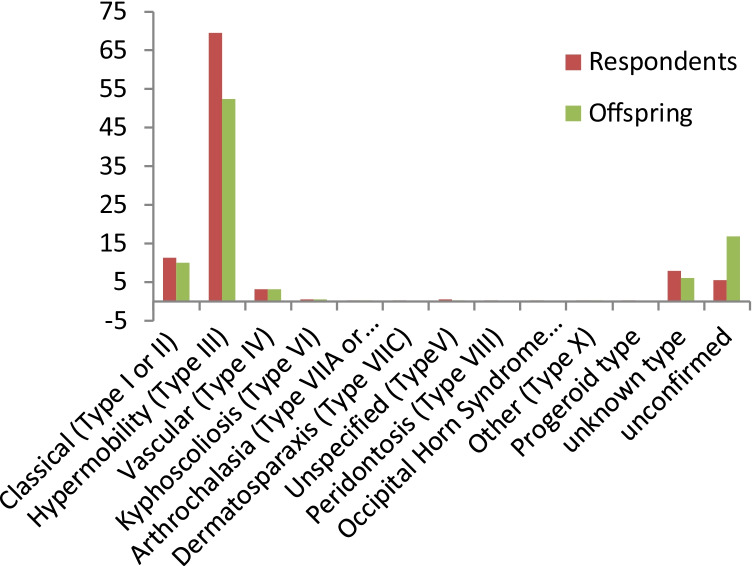
Percent of EDS subtypes reported for respondents and offspring. All 10 listed EDS subtypes were represented by the 1320 respondents, with type VI through type X each accounting for < 1% of the total. In the offspring diagnosis pattern, type VIIC, types VII and IX, and progeroid were not represented

The majority of respondents (56.5%) were 18–40 years old, followed by 38.3% between 41 and 60 years old, 3.7% > 60 years old, and 1.6% < 18 years old. Of those respondents, 87.9% have EDS. Respondents’ diagnoses included all types of EDS: type III—hypermobility was the most common (69.5%), followed by type I or II—classical (11.3%), and type IV—vascular (3.2%) (Fig. 1). Respondents’ offspring’s diagnoses displayed a similar diagnosis pattern with type III being the most common (52.4%), followed by type I or II (10%), and type IV (3.2%) (Fig. 1).

Of the 1321 respondents with EDS, 21.2% reported having a mother with EDS, 33.4% had a mother without EDS, while 45.2% reported their mother’s EDS status as “unknown.” Of the 1068 pregnancies reported by respondents with EDS, 52.4% had infants with EDS, while 28.5% had infants without EDS. Respondents without EDS reported 191 pregnancies, with 47.6% resulting in infants with EDS, and 40.3% resulting in infants without EDS.

**Fig. 2:**
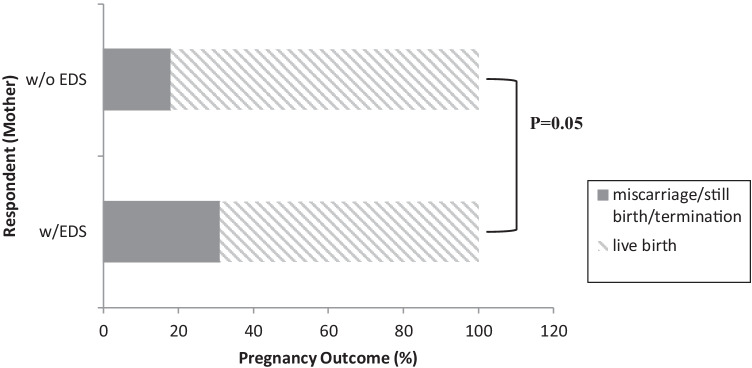
The percent of pregnancies reported that resulted in live versus non-live births based on the maternal EDS status is presented. The percent of pregnancies reported that resulted in live births for EDS mothers versus non-EDS mothers was 68.9% and 82.1% respectively. Pregnancies that resulted in miscarriage/stillbirth/termination for EDS mothers versus non-EDS mothers as 31.0% and 17.9% respectively. (*p* = 0.05)

When pregnancy outcomes for respondents with versus without EDS were compared, there were significantly more (*p* < 0.005) outcomes such as miscarriage, stillbirth, and termination of pregnancy reported by mothers with EDS, than by mothers without EDS regardless of the offspring’s EDS status (Fig. 2).

Respondents and offspring were categorized as having the diagnosis of EDS or not (EDS or non-EDS). Thus, three groups emerged: EDS mother/EDS infant, EDS mother/non-EDS infant, and non-EDS mother/EDS infant. Poor pregnancy outcomes in these three groups are presented in Table 1. The EDS mother/EDS infant group reported higher percentages of birth complications and treatment for preterm birth during pregnancy than the EDS mother/non-EDS infant and non-EDS mother/EDS infant group, while the non-EDS mother/EDS infant group exhibited a higher rate of preterm birth. Mean gestational age at preterm birth was significantly different amongst groups.

**Table 1:** Proportions of pregnancy outcomes for three maternal/fetal EDS groups

Categories	EDS mother/EDS infant *n* = 559 (%)	EDS mother/non-EDS infant *n* = 304 (%)	Non-EDS mother/EDS infant *n* = 91 (%)	*P* value
Birth complications	447 (80)	221 (72.2)	65 (71.4)	0.024
Cesarean delivery	178 (31.8)	82 (27)	33 (36.2)	NS
Treatment for PTB	165 (29.5)	59 (19.4)	19 (20.9)	0.003
PTB	173 (30.9)	72 (23.7)	33 (36.2)	0.008
Mean gestational age @ PTB	33 ^5/7^ (23–36)	34 ^4/7^ (24–36)	33 ^6/7^ (26–36)	0.008

*PTB*, preterm birth; *NS*, not statistically significant

Next, to identify the association of fetal versus maternal diagnosis of EDS to poor pregnancy outcomes, two groups were compared: one based on maternal diagnosis (EDS versus non-EDS), and the other based on fetal diagnosis (EDS versus non-EDS). When mothers with EDS were compared to mothers without EDS, no difference was observed between the groups for proportions of birth complications, cesarean delivery, treatment for PTB, occurrence of PTB, or mean gestational age. When the groups were compared based on the infant’s diagnosis (EDS versus non-EDS), an increased rate for each category except cesarean delivery was observed in the infants with EDS compared to infants without EDS (Table 2).

**Table 2:** Proportions of pregnancy outcomes by fetal EDS groups

Categories	Yes EDS *n* = 650 (%)	No EDS *n* = 340 (%)	Relative Risk (95% CI)	*P* value
Birth complications	512(78.8)	221 (72.7)	1.08 (1.00–1.11)	0.038
Cesarean delivery	211 (32.46)	82 (27.0)	1.20 (0.97–1.49)	NS
Treatment for PTB	175 (26.9)	59 (19.4)	1.39 (1.07–1.80)	0.012
PTB	206 (31.7)	72 (23.7)	1.34 (1.06–1.69)	0.011
Mean gestational age @ PTB	33 ^5/7^ (23–36)	34 ^4/7^ (24–36)		0.002

*PTB*, preterm birth; *NS*, not statistically significant

The ten most frequently reported categories of ante-, intra-, and post-partum complications associated with pregnancies resulting in preterm birth are presented in Table 3. Of these, the occurrence of PPROM, a subcategory of PTB, was significantly different between the three mother/infant groups. When the infant groups with EDS and without were compared, no significant differences between the categories of ante-, intra-, and post-partum complications were revealed. The comparison between mothers with or without EDS revealed a significantly (*p* = 0.007) higher percentage of PPROM in mothers without EDS compared to mothers with EDS (relative risk = 0.36, with % CI 0.18–0.72).

**Table 3:** The 10 most common ante-, intra-, and post-partum complications resulting in preterm birth reported based on mother/infant EDS groups

Complication	EDS mother/EDS infant *n* = 128 (%)	EDS mother/non-EDS infant *n* = 48 (%)	Non-EDS mother/EDS infant *n* = 18 (%)	*P* value
PE, hypertension, HELLP	24 (18.8)	12 (25.0)	6 (33.3)	NS
Placental issues (previa, accreta, hematoma, abruption)	21 (16.5)	4 (8.4)	1 (5.6)	NS
PPROM	19 (14.8)	6 (12.5)	7 (38.9)	0.025
Symphysis pubis dysfunction, Torn sacroiliac ligament	19 (14.8)	6 (12.5)	0 (0.0)	NS
Pre/intra/postpartum bleeding	12 (9.4)	6 (12.5)	1 (5.6)	NS
Other organ issues (*cholestasis, kidney failure/stones/infection, polyarthralgia*	11 (8.6)	1 (2.1)	0 (0.0)	NS
Gestational diabetes	7 (5.5)	1 (2.1)	1(5.6)	NS
IUGR	7 (5.5)	0 (0.0)	0 (0.0)	NS
Uterine anatomical abnormalities (septate and underdeveloped)	2 (1.6)	0 (0.0)	0 (0.0)	NS
Uterine rupture	2 (1.6)	0 (0.0)	0 (0.0)	NS

Data are *n* (%). Abbreviations: *EDS* Ehlers-Danlos syndrome, *PE* preeclampsia, *HELLP* a syndrome characterized by hemolysis, elevated liver enzymes, low platelet count, *PPROM* preterm premature rupture of membranes, *IUGR* intrauterine growth restriction. *NS* not statistically significant

## Conclusions

This study is the first to describe self-reported gestational outcomes in pregnancies complicated by Ehlers-Danlos syndrome.

Our study revealed that women with EDS report more pregnancy outcomes such as miscarriage, stillbirth, and abortion compared to women without EDS who give birth to children with EDS. Previous studies have also shown a higher rate of spontaneous abortions, miscarriages, and stillbirths in the 3 most common EDS subtypes compared to the general population [4, 5, 7, 8]. Other studies that focused on 2 of the 3 most common EDS subtypes (hypermobility and classical) concluded that pregnancies involving women with those EDS subtypes do not have an increased risk of adverse pregnancy outcomes [3, 6, 9, 16]. Our study expanded the EDS subtypes represented to include all 3 common subtypes, as well as rare types, resulting in a more complete representation of EDS-related adverse pregnancy outcomes. Although the number of respondents with less common EDS subtypes is low in our study, we believe it is important for them to be included when discussing/evaluating pregnancy outcomes in EDS-related pregnancies.

The acquisition of data regarding both the maternal and infant EDS status allowed conclusions to be drawn regarding maternal versus fetal association to the observed outcomes. Our data demonstrate that the percentage of birth complications as well as the percentage of pregnancies treated for preterm birth is highest in pregnancies in which both the mother and the infant have EDS, and the gestational age at preterm birth is lowest in this group, while the rate of preterm birth is highest in mothers without EDS who have infants with EDS. A possible explanation for these findings is that pregnancy complications unrelated to preterm birth may be more common in women with EDS, and treatment for preterm birth may be higher as they may be observed more closely during pregnancy than women without EDS. This closer clinical observation and rate of intervention to prevent preterm birth in EDS mothers is likely not equivalent to that experienced by non-EDS mothers, since they may not be considered at risk if it is not known that they are carrying an EDS infant. EDS-related preterm birth is likely driven by fetal contribution. Early case studies have similarly concluded that infants with EDS were more likely to be born prematurely than their siblings without EDS [12, 17].

Previous studies have shown that phenotypically healthy women with a history of recurrent PPROM have connective skin abnormalities reminiscent of EDS [18], that premature infants with EDS are more likely to have been born prematurely due to PPROM than the general population of premature infants [12], and in a Dutch population, women without EDS were more likely to deliver their infant with EDS preterm than women with EDS [3]. Our study confirms these findings and shows that PPROM as a complication associated with preterm birth is more likely to occur in non-EDS mother/EDS infant pregnancies than in the other two pregnancy groups, and accounts for close to 40% of the preterm births in the non-EDS mother/EDS infant pregnancies, slightly higher than the expected occurrence of preterm births due to PPROM in the overall population [19, 20]. Further analysis revealed that PPROM was reported more frequently if the mother did not have EDS, because in this group, all infants have EDS, compared to the maternal EDS group, in which not all infants have EDS. One explanation for these observations is that the fetal membranes are of fetal origin and thus a reflection of the fetus’ genetic makeup and not the mother’s. Thus, fetal membranes that likely display abnormal connective tissue extracellular matrix similarly to the somatic connective tissue of patients diagnosed with EDS may be more likely to rupture, leading to PPROM and preterm birth. Our mouse model of EDS, the biglycan decorin double knockout mouse, displays preterm birth, as well as morphologic and signaling abnormalities of the fetal membranes [14, 15]. Conversely, no difference in PPROM occurrence was noted between EDS and non-EDS infant groups in our study. We believe that the lack of increased risk of PPROM in the EDS infant (with EDS and non-EDS mothers) group compared with the non-EDS infant (only with EDS mothers) group may be due to the fact that mothers with EDS reported an increased risk of pregnancy complications that may interrupt the pregnancy prior to the time at which PPROM would have occurred.

While previous studies have indicated an association, our study is the first to demonstrate the importance of the infant EDS genotype compared to the maternal genotype in PPROM. Another important question, which is beyond the sample size of this study, is whether the risk of PPROM is dependent on the EDS subtype.

Our data also shows that mean gestational age at PTB is associated with the EDS status of the infant but not the EDS status of the mother. This again suggests that PTB may be driven by fetal EDS, possibly related to the altered composition of the fetal membranes secondary to the genetic abnormalities associated with EDS. However, a caveat to these findings is that the differences found in the mean gestational age may not be clinically relevant.

Our survey was conducted prior to the revised EDS classification in 2017 [1], so the previously recognized subtypes were used in our survey [21]. Since hypermobile, classical, and vascular subtypes are the most common forms of EDS in both formal classifications, and these three subtypes also represent the three largest diagnosis groups reported in our survey, these results are nonetheless relevant.

While the data gathered from this survey are based on participants’ recollection of past events, previous studies have shown that maternal recall of obstetric data is a valid method for data collection, and that a correlation exists between the severity of the obstetric complication and recall accuracy [22–24]. Although data was not collected on the elapsed time between delivery and survey completion, a previous study by Tomeo et al. concluded that due to the salient nature of pregnancy, maternal recall was accurate even 30 or more years after delivery [25].

This study describes the outcomes of pregnancies affected by EDS, as well as identifying the key concept that outcomes differ depending on whether it is the mother, the infant, or both who have EDS. These insights into maternal versus fetal association to adverse pregnancy outcomes in pregnancies complicated by EDS can further guide physicians in educating, managing, and treating pregnant women with EDS-related pregnancies.

## Supplementary Information

Below is the link to the electronic supplementary material.

Supplementary file1 (24.3 KB)
