# Supplementary material for: Association of Maternal Versus Fetal Ehlers-Danlos Syndrome Status with Poor Pregnancy Outcomes
Source: Reprod Sci. 2022 Jun 8;29(12):3459–64. doi: 10.1007/s43032-022-00992-1 (PMC9734202; doi:10.1007/s43032-022-00992-1)
Supplement: Supplementary file 1 — Supplementary file1 (24.3 KB) [file 43032_2022_992_MOESM1_ESM.docx]

Ehlers-Danlos Syndrome and Birth Complications Survey

Women who have Ehlers-Danlos Syndrome or whose infant has Ehlers-Danlos Syndrome are at increased risk of birth complications such as preterm birth. However, not much is known about these complications. With this survey we hope to investigate the relationship between Ehlers-Danlos Syndrome and preterm birth. Thank you very much for your help with this study!

1. **What is your current age in years?**
2. <18
3. 18-40
4. 41-60
5. >60
6. **Do you have Ehlers-Danlos Syndrome?**
7. Yes
8. No
9. **At what age were you diagnosed with Ehlers-Danlos Syndrome?**

|  |
| --- |

1. **Which of the following best describes your diagnosis?**
2. Classical (Type I or II)
3. Hypermobility (Type III)
4. Vascular (Type IV)
5. Kyphoscoliosis (Type VI)
6. Arthrochalaisia (Type VIIA or VIB)
7. Dermatosparaxix (Type VIIC)
8. Unspecified (Type V)
9. Peridontosis (Type VIII)
10. Occipital Horn Syndrome (Type IX)
11. Other (Type X)
12. Progeroid type
13. I have Ehlers-Danlos syndrome, but I’m not sure what type
14. I may have Ehlers-Danlos syndrome, but the diagnosis has not been confirmed
15. Other (please specify)

|  |
| --- |

1. **Is your Ehlers-Danlos genetically confirmed?**
2. Yes (please specify the genetic mutation)
3. No

Please specify genetic mutation.

|  |
| --- |

1. **Were there any complications for you or your mother during the pregnancy or your birth?**
2. Yes (please specify)
3. No

Please specify complications if known.

|  |
| --- |

1. **Were you born via vaginal birth or Cesarean section?**
2. Vaginal birth
3. Cesarean section
4. Additional comments

|  |
| --- |

1. **Did your mother have Ehlers-Danlos Syndrome?**
   1. Yes (Please specify type if known)
   2. No
   3. I don’t know

Please specify type if known

|  |
| --- |

1. **Were you born prematurely ( at less than 37 weeks of pregnancy)?**
2. Yes
3. No
4. **At what gestational age were you born?**
5. 23 weeks
6. 24 weeks
7. 25 weeks
8. 26 weeks
9. 27 weeks
10. 28 weeks
11. 29 weeks
12. 30 weeks
13. 31 weeks
14. 32 weeks
15. 33 weeks
16. 34 weeks
17. 35 weeks
18. 36 weeks
19. Other (please specify)

|  |
| --- |

1. **Please specify the reason for preterm birth, if known (Preeclampsia, abruption, preterm premature rupture of membranes, etc)?**

|  |
| --- |

1. **Are you male or female?**
2. Male
3. Female

If response is (a); survey ends here.

If response is (b); survey continues to next question.

1. **Have you ever been pregnant?**
2. Yes
3. No

If response is (a) yes; survey continues to next question.

If response is (b) no; survey ends here.

1. **What was the outcome of your first pregnancy?**
2. Miscarriage, abortion or stillbirth
3. Live birth

If response is (a), survey automatically jumps to Question #24.

If response is (b), survey continues to next question.

1. **What is the current age of this child?**

|  |
| --- |

1. **At what gestational age was he/she born?**
2. 23 weeks
3. 24 weeks
4. 25 weeks
5. 26 weeks
6. 27 weeks
7. 28 weeks
8. 29 weeks
9. 30 weeks
10. 31 weeks
11. 32 weeks
12. 33 weeks
13. 34 weeks
14. 35 weeks
15. 36 weeks
16. 37 weeks or higher
17. Other (Please specify)

|  |
| --- |

1. **Were there any complications for you or your baby during pregnancy or birth?**
2. Yes (please specify)
3. No

Please specify if there were any complications.

|  |
| --- |

1. **Was the baby born vaginal birth or Cesarean section?**
2. Vaginal birth
3. Cesarean section

Additional comments

|  |
| --- |

1. **During your pregnancy did you receive a treatment to prevent preterm birth?**
2. Yes (Please specify the treatment)
3. No

Please specify the treatment

|  |
| --- |

1. **Does this child have Ehlers-Danlos Syndrome?**
2. Yes
3. No

If response is (a)yes; survey continues to next question.

If response is (b) no; survey automatically jumps to Question 24.

1. **Which of the following best describes his/her diagnosis?**
2. Classical (Type I or II)
3. Hypermobility (Type III)
4. Vascular (Type IV)
5. Kyphoscoliosis (Type VI)
6. Arthrochalaisia (Type VIIA or VIB)
7. Dermatosparaxix (Type VIIC)
8. Unspecified (Type V)
9. Peridontosis (Type VIII)
10. Occipital Horn Syndrome (Type IX)
11. Other (Type X)
12. Progeroid type
13. My child has Ehlers-Danlos syndrome, but I’m not sure what type
14. My child has Ehlers-Danlos syndrome, but the diagnosis has not been confirmed
15. Other (please specify)

|  |
| --- |

1. **Is his/her Ehlers-Danlos Syndrome genetically confirmed?**
2. Yes
3. No

Please specify the genetic mutation.

|  |
| --- |

1. **Does your child’s father have a diagnosis of Ehlers-Danlos Syndrome?**
2. Yes (Please specify type if known)
3. No
4. I don’t know

Please specify type of EDS if known.

|  |
| --- |

1. **Have you had another pregnancy?**
2. Yes
3. No

If response is (a)yes; survey continues to next question.

If response is (b)no; survey ends here.

1. **What was the outcome of this pregnancy?**
2. Miscarriage, abortion, stillbirth
3. Live birth

If response is (a); survey automatically jumps to Question 24.

If response is (b); survey automatically jumps to Question 15.
